# Head and Neck Paragangliomas in the Czech Republic: Management at the Otorhinolaryngology Department

**DOI:** 10.3390/diagnostics12010028

**Published:** 2021-12-23

**Authors:** Anasuya Guha, Martin Chovanec

**Affiliations:** Department of Otorhinolaryngology, University Hospital Kralovske Vinohrady, 3rd Faculty of Medicine, Charles University, 10034 Prague, Czech Republic

**Keywords:** HNPGLs, jugular paragangliomas, incidentaloma, Shamblin’s classification, Fisch’s classification, facial nerve palsy, cranial nerve dysfunction, otorhinolaryngology

## Abstract

Head and neck paragangliomas (HNPGLs) are rare neuroendocrine tumors, comprising only 3% of all head and neck tumors. Early diagnosis forms an integral part of the management of these tumors. The two main aims of any treatment approach are long-term tumor control and minimal cranial nerve morbidity. The scope of this article is to present our case series of HNPGLs to stress most important clinical aspects of their presentation as well as critical issues of their complex management. Thirty patients with suspected HNPGLs were referred to our otorhinolaryngology clinic for surgical consultation between 2016–2020. We assessed the demographical pattern, clinicoradiological correlation, as well as type and outcome of treatment. A total of 42 non-secretory tumors were diagnosed—16.7% were incidental findings and 97% patients had benign tumors. Six patients had multiple tumors. Jugular paragangliomas were the most commonly treated tumors. Tumor control was achieved in nearly 96% of operated patients with minimal cranial nerve morbidity. Surgery is curative in most cases and should be considered as frontline treatment modality in experienced hands for younger patients, hereditary and secretory tumors. Cranial nerve dysfunction associated with tumor encasement is a negative prognostic factor for both surgery and radiotherapy. Multifocal tumors and metastasis are difficult to treat, even with early detection using genetic analysis. Detecting malignancy in HNPGLs is challenging due to the lack of histomorphological criteria; therefore, limited lymph node dissection should be considered, even in the absence of clinical and radiological signs of metastasis in carotid body, vagal, and jugular paragangliomas.

## 1. Introduction

Extra-adrenal pheochromocytomas may arise in any portion of the paraganglion system; however, they most commonly occur below the diaphragm [1]. The second most common site is located in the head and neck and are classified by the World Health Organization [2,3]. Head and neck paragangliomas (HNPGLs) are rare neuroendocrine tumors, representing 3% of all head and neck tumors [4]; only 1–3% of HNPGLs are functional or catecholamine secreting [5]. These tumors can be sporadic or as part of genetic syndromes, and it has also become apparent that about 30–35% of sporadic tumors are due to a germline mutation, otherwise known as ‘occult familial’ cases [6,7,8]. Genes associated with the *succinate dehydrogenase* (*SDH_x_*) complex are most often seen mutated in these types of tumors, more commonly the *SDHD* followed by the *SDHB* subunits [6]. Patients with HNPGLs mostly become symptomatic between the fourth and seventh decade; it should be mentioned that patients with sporadic tumors usually present older than 40 years [6,9]. They also show a female predominance of 3–4:1 [9]. The site of origin defines the name given to HNPGLs [6]. These paragangliomas (PGLs) arise preferentially from the carotid body (carotid body paragangliomas; CBPGLs), the jugular bulb (jugular paragangliomas; JPGLs), along the tympanic branch of the glossopharyngeal nerve (tympanic paragangliomas; TPGLs), and the vagus nerve (vagal paragangliomas; VPGLs) [3]. The diagnostic work-up for such patients is an elaborate method and therefore a multidisciplinary approach is instituted for successful management. After completion of a thorough medical and family history as well as clinical examination, three key investigations should be done. These include biochemical tests (catecholamine and chromogranin A levels), radiological evaluation (ultrasonography of the neck followed by both anatomical [MRI or CT scan] for localization as well as functional imaging [PET-CT] to rule out multifocal tumors as standard practice) and genetic mutation analysis (following patient consent). Carotid body tumors or CBPGLs represent the most common tumor type (up to 60%) and typically present with a painless, slowly enlarging mass in the lateral part of the neck. Dysphagia, dysphonia, dysarthria (deficits of the IX-XI, XII cranial nerves), as well as Horner syndrome and syncope may be seen with increasing tumor size. Bilateral tumors are seen in 10% of patients and malignancy in about 6–12% [10,11,12,13]. The Shamblin classification (MRI-based) is used to assess the vascular morbidity of surgical resection. This describes the relationship of the tumor to the external, internal, and common carotid arteries [14]. Other frequently detected HNPGLs include jugulotympanic (<35–40%) and vagal (<5%). Patients with jugulotympanic tumors range from asymptomatic to presenting with pulsatile synchronous tinnitus, conductive hearing loss, lower cranial nerve deficits of mainly IX and X, and even occasionally with Vernet and Villaret syndromes [12,15]. Malignancy rate of jugulotympanic PGs is reported in 5.1% [16]. The Fisch’s classification (CT-based) is used to classify these tumors as it indicates the extent of temporal bone destruction [5]. Whilst with vagal tumors, 75% of patients have a painless lateral neck mass, 100% with pulsation, 55% have pharyngeal mass, 28–54% suffer hoarseness of voice, pulsatory tinnitus, other lower cranial nerve deficits (17% in XII, 11% in IX, 6% in X), and Horner syndrome [17]; risk of malignancy is 6–19% [18]. Extremely rare cases of paragangliomas can be found in the nose or the paranasal sinuses, orbit, larynx, parotid gland, cervical sympathetic chain, thyroid, parathyroid gland, and esophagus [9,19,20,21,22,23,24,25]. Sinonasal paragangliomas mostly affect the middle turbinate and ethmoid sinus and lead to obstruction and epistaxis [19]. Malignancy rate of 24% in nasal and paranasal PGs is highest amongst all types of HNPGLs [16]. Laryngeal PGLs, with 82% arising from the supraglottic larynx, manifest clinically with hoarseness [20]. It should also be noted that due to the nature of HNPGLs, incidental finding of these tumors is not an uncommon phenomenon. In this study, we analyzed in detail the pattern of tumors, including management outcome amongst our cohort of patients.

## 2. Materials and Methods

### 2.1. Subject Selection Criteria

Inclusion: All patients with suspected or diagnosed unilateral or multiple HNPGLs (benign or malignant tumors) between the ages of 18–85 years old referred to our otorhinolaryngology department for surgical consultation between October 2016 and October 2020. These patients were either referred from specialists such as endocrinologists, neurosurgeons, or from primary care otorhinolaryngological practices.

### 2.2. Management Protocol

A multidisciplinary approach was adopted in all the patients. After detailed medical and family history, patients underwent complete standard otorhinolaryngological examination including audiometry and clinical investigations.

#### 2.2.1. Characterization of Tumors Based on Clinicoradiological Investigations

- Biochemical examination: Plasma metanephrine and normetaphrine levels were measured to ascertain secretory status of tumors. Chromogranin A, a tumor marker for pheochromocytomas/paragangliomas, was also determined for all patients.
- Analysis using imaging techniques: Following ultrasound of the neck, CT or MRI including CT- or MR-angiography according to the localization, Shamblin’s classification was employed to classify the extent of CBPGLs, whilst Fisch’s classification was used for vagal and jugular PGLs. Furthermore, 18F-FDOPA PET/CT scan was done for all patients with multiple tumors, suspected metastases, and/or progression of disease on follow-up.
- Germline genetic testing: According to our protocol, all patients who consented underwent genetic examination. Peripheral blood samples were taken for DNA extraction and analysis. Firstly, preliminary germline genetic testing using Polymerase Chain Reaction (PCR) Sanger sequencing was done using the Mutation Surveyor^®^ (Carolina Biosystems, Czech Republic) to exclude *SDHD* followed by *SDHB* mutation; subsequently, patients were referred for genetic counselling to complete Next Generation Sequencing (NGS) examination. NGS utilizing NextSeq 500 (Illumina^®^, San Diego, CA, USA) was used to analyze 123 standard panel genes for pheochromocytoma/paraganglioma. In certain cases, NGS examination was already completed before referral to us. Relevant family members of index patients with positive genetic mutation were also invited for genetic carrier testing.

#### 2.2.2. Institutional Ethics Board Statement

All procedures performed in studies involving human subjects were in compliance with the Helsinki declaration and further in accordance with local ethical guidelines of the institutional ethical committees of Charles University, Prague, Czech Republic.

Informed consent was obtained for all patients undergoing intervention according to the hospital regulations, institutional guidelines of Charles University, and those defined by the practice codes of the Ministry of Health of the Czech Republic. Additionally consent was obtained for genetic testing.

#### 2.2.3. Treatment Plan

##### Therapeutic Approaches

Finally, a treatment plan was devised in each case after thorough consultation. A decision on interventional therapy (surgery or radiotherapy) or observation was ascertained. The decision depended on clinical factors such as the age and medical status of the patient, size, localization, multiplicity of the tumor, previous interventions, and choice of the patient.

In our study cohort, surgical exploration or conservative approach with the intention of ‘wait and scan’ were considered. Wait and scan is also carried out on the basis of yearly MRI scan.

##### Protocol for Surgery

Decision for surgery included the age and comorbidity of the patient, cranial nerve functional status, clinicoradiological classification, size and multiplicity of tumor, and progression of disease.

- Preoperative embolization

All patients who underwent surgery had preoperative embolization under image guidance. This represents standard protocol in all but small CBPGLs (Shamblin I) and TPGLs (Fish A) to reduce the risk of perioperative morbidity and mortality. Therefore, patients who were assigned to surgery underwent preoperative embolization of the feeding arteries during digital subtraction angiography. We used gelfoam for small tumors and Onyx (ethylene vinyl alcohol copolymer) for larger ones. In patients with either advanced CBPGLs (Shamblin II and III) or JGPGLs (Fish C3 and C4), we also adopted strategy of preoperative reinforcement of carotid artery with stenting six weeks before the surgery.

- Surgical techniques

Please refer to sections ‘results’ and ‘discussion’. Tumor samples were collected for future analysis of somatic mutation.

- Postoperative care

Postoperative corticosteroid therapy was administered in patients undergoing infratemporal fossa type A approach with anterior facial nerve rerouting for a period of 14 days. Cranial nerve dysfunction was analyzed immediately, 2 weeks, 1, 3, and 6 months postoperatively, followed by yearly reviews. Local tumor control was assessed over a period of 1 to 4 years follow-up using MRI and PET/CT according to protocol. Imaging was done on an annual basis, that is MRI in solitary tumors and PET/CT in hereditary, multiple, and malignant tumors.

We evaluated the gender distribution, symptomatology, clinical and laboratory tests, localization and classification of PGLs, as well as type and outcome of treatment.

## 3. Results

A total of 30 patients (36.7% males, 63.3% females) of 34–80 years of age were diagnosed with HNPGLs. All patients were of central European origin (28 patients were Czech, patient No. 5 was Hungarian, patient No. 27 was Polish). Only 2 patients had a positive family history of HNPGLs (Table 1). The commonest symptoms were hearing difficulties, tinnitus, and painless neck mass (Table 2). Four patients were diagnosed with incidental HNPGLs on imaging studies and one on preoperative findings during neck surgery.

### 3.1. Analysis of Audiometric Examination

Amongst those with JPGLs, three had mixed hearing loss, three had sensorineural hearing loss/deafness and one had conductive hearing loss; all patients with TPGLs had conductive hearing loss. Other patients had normal hearing.

### 3.2. Characteristics of PGLs and Genetic Results

Amongst 30 patients, 24 had unilateral and 6 had bilateral/multiple tumors (Table 1). A total of 42 HNPGLs were found (24 were left-sided, 18 right-sided). According to localization of tumors, we found 11 CBPGLs, 13 JPGLs, 8 TPGLs, and 10 VPGLs.

#### 3.2.1. Localization of Head and Neck Tumors

Unilateral PGLs

##### Carotid Body

A total of 4 patients were diagnosed with single carotid body tumors. All were of Czech origin. Patient No. 4 had a sister with carotid body tumor. The most common symptom reported in patients with carotid body tumors was painless neck swelling on the affected side. Patient No. 2 was asymptomatic and the tumor was an incidental finding (Table 2). Two patients were negative for any germline mutation, the other 2 were negative for PCR *SDHD* mutation.

##### Jugular

Eight patients presented with jugular paragangliomas. The youngest patient was of Hungarian origin. All patients complained of hearing difficulties and 3 patients had pulsatory tinnitus. Patients 7 and 10 presented with varied signs of lower cranial nerve dysfunction (dysphonia, dysphagia, and dysarthria); the latter had a more severe dysfunction. Patient No. 9 had facial nerve paralysis. All patients had cranial nerve VIII paralysis. Patients 6, 7, 8, and 11 tested negative for germline mutation and the rest were *SDHD* negative on PCR examination.

##### Tympanic

Out of 7 patients diagnosed with tympanic tumors, one was a second recurrence. All complained of hearing difficulty and objectively had conductive hearing loss; only 3 had pulsatory tinnitus. Patient No. 15 also had an incidental finding of frontoparietal meningioma on MRI findings. All patients tested negative for *SDHD* mutation.

##### Vagal

Five patients were diagnosed with unilateral vagal tumors. One patient had an incidentaloma (Table 2), diagnosed on follow-up ultrasound scan of the neck for hypothyroidism and another was diagnosed during preoperative findings in suspected metastasis of thyroid gland. All patients presented with painless lump in the neck. Patients No. 20 was *SDHD* negative and 21 tested positive for germline *SDHB* mutation, the rest did not undergo genetic examination.

Multiple or Bilateral PGLs

**Figure 1:**
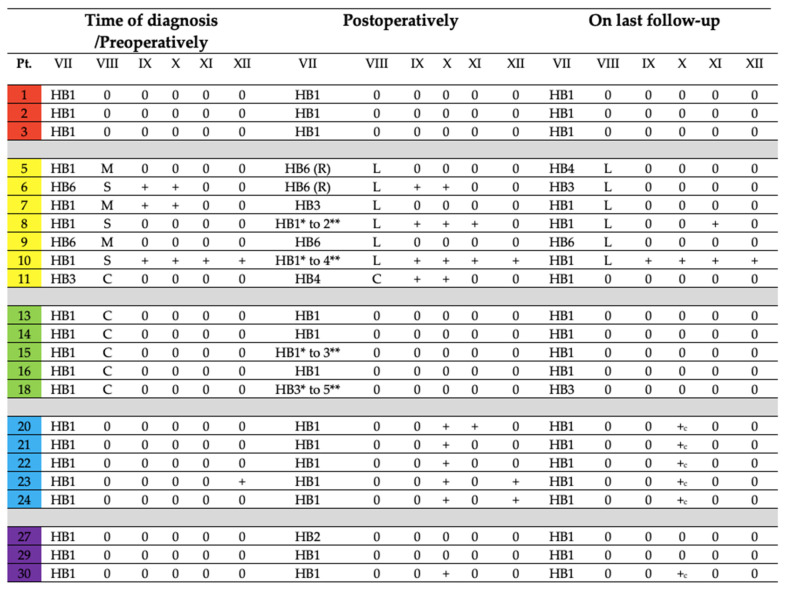
Vagal PGL on (**a**) MRI of the neck (coronal view) and (**b**) 18F-FDOPA PET-CT as well as (**c**) retroperitoneal PGL on 18F-FDOPA PET-CT seen in a patient with *SDHB* mutation.

Results from a previous publication are being included here [26]. Six patients were diagnosed with 18 multiple benign HNPGLs. Patient No. 25 had bilateral CBPGLs (Shamblin III on the left, Shamblin I on the right) and cyanotic congenital heart disease. Patient No. 26 had very advanced disease and confirmed dysfunction of VII-XII cranial nerves. Only the patient of Polish origin (patient No. 27) had a positive history of VPGL on her father’s side of the family. Patient No. 28 had incidental findings of left-sided vagal and jugular tumors (Figure 1), which were stable, with minimal progress over the last 10 years. The 51-year old patient in our series was also diagnosed incidentally with 5 HNPGLs as he presented with intractable otorrhagia. The last patient developed JPGL on follow-up. *SDHD* mutation was found in patients 26, 27, and 30 whilst *SDHB* mutation was detected in patient No. 28; the other 2 were negative on NGS genetic examination.

Carrier status of relatives were not determined since the family members did not consent to the examination.

#### 3.2.2. Localization of Paragangliomas below the Neck

Out of 6 patients with multiple benign HNPGLs, 3 were diagnosed with other paragangliomas on 18F-FDOPA PET/CT imaging studies [26]. Patients No. 27 and 28 (Figure 1) were diagnosed with retroperitoneal PGL, whilst patient No. 26 with anterior mediastinal PGL.

No signs of metastasis to regional lymph nodes, bones, lungs, and liver were detected in any of our patients.

#### 3.2.3. Biochemical Activity of Tumors

Plasma metanephrine (0.140–0.540 nmol/L) and normetanephrine (0.130–0.790 nmol/L) levels were within physiological limits for all patients, hence all tumors were considered non-secretory.

All had normal levels for Chromogranin A (0–85 ng/mL), a tumor marker for pheochromocytomas/paragangliomas, except for patients No. 7 (156.5 ng/mL), 26 (231.4 ng/mL) and 27 (224.7 ng/mL).

### 3.3. Treatment Modality and Outcome

After completion of all examinations, patients were either allocated to surgery or ‘wait and scan’ approach. A total of 23 patients (20 with unilateral single tumors, 3 with multiple HNPGLs) underwent surgery (Table 1). One patient died from complications of very advanced disease and the rest were allocated to ‘wait and scan’ method using MRI scan.

#### 3.3.1. Surgery

All paragangliomas were operated by us in our department. No other specialists were required for surgical intervention.

##### Preoperative Embolization

**Figure 2:**
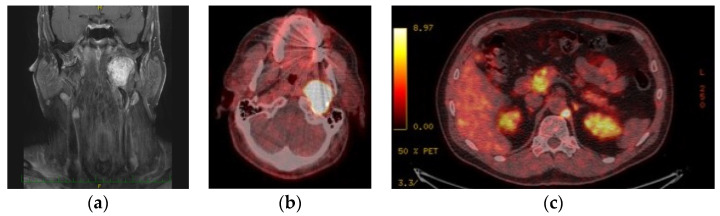
Status of cranial nerve function in patients who underwent surgery. Color key: Red = CBPGL; Yellow = JPGL; Green = TPGL; Blue = VPGL; Purple = Multiple PGLs(HB: House-Brackmann; M: Mixed Hearing Loss; C: Conductive Hearing Loss; S: Sensorineural Hearing Loss; R: Reconstructed; L: Labyrinthectomy leading to deafness; *: early postoperative function; **: 10 week postoperative function; +_c_: compensated nerve dysfunction).

No complications were encountered in all patients following embolization. However, in two patients with extensive jugular paragangliomas, deterioration of cranial nerve status was seen following intervention. Patient No. 6 had lower cranial nerve as well as facial nerve dysfunction and patient No. 10 had VIII–XII nerve palsy (Figure 2). In both of these patients, encasement of affected cranial nerves with tumor was the primary intraoperative finding.

##### Surgical Approaches

Three out of 4 patients with single CBPGLs (Shamblin II) underwent surgery (level II neck dissection and tumor excision). Perioperative and postoperative complications such as stroke and hemorrhage or hematoma that needed revision were not seen; only 1 patient developed ‘First Bite’ syndrome postoperatively, which resolved with time. No post-operative cranial nerve dysfunction was noted.

Seven patients with JPGL underwent removal of the tumor via the Infratemporal fossa type A (IFTA) approach and level II selective neck dissection (SND). In both patients with preoperative facial nerve palsy, intraoperative finding of nerve invasion by tumor was seen; the facial nerve was reconstructed. Patient No. 7 with Fisch C3 Di3 had palsy of the facial nerve House–Brackmann (HB) III postoperatively, which improved after rehabilitation. The surgery was planned as a two-step approach with removal of intradural tumor as second phase. However as the intradural tumor shrunk, we adopted a ‘wait and scan’ policy and the residual intradural portion of tumor was stable and the patient was asymptomatic. Patient No. 8 had dysfunction IX-XI after surgery, but well compensated. Patient No. 10 had persistence of dysfunction of cranial nerves VIII-XII with resolved facial nerve palsy. Lastly, patient No. 6 who had worsened postembolization cranial nerves VII (HB VI), IX, and X palsy due to tumor infiltration additionally underwent facial nerve reconstruction. This improved facial nerve paralysis to HB III. The dysfunction of cranial nerves IX and X was also resolved. She was also referred for voice rehabilitation and electrostimulation of muscles.

Five patients with TPGLs had extirpation of the paraganglioma; patient No. 18 additionally underwent level II SND; two patients with Fisch C tumors had postoperative facial nerve palsy, only one had permanent dysfunction (Figure 2).

Those diagnosed solely with VPGLs underwent extirpation of the tumor and level II SND, excepting patient No. 24, who had revision SND levels II-V because of neck lymphadenopathy suspicious of neck metastases. All had vagal nerve dysfunction postoperatively, but compensated over time (Figure 2). Only 1 patient had cranial nerve XI dysfunction and two had hypoglossal nerve palsy, which improved after rehabilitation.

Three patients with multiple HNPGLs were operated on. Patient No. 29 subsequently underwent emergency embolization of the TPGL. He also underwent surgery for the other tumors in a step-wise manner.

##### Histopathology Results

On histopathology examination, paragangliomas were confirmed on the basis of the classic ‘Zelballen’ pattern. About 95.7% of all surgically removed tumors were confirmed as benign with no metastatic disease on histopathology examination. Tumor metastasis in the otherwise non-enlarged lymph node was found only with patient No. 8, who had a jugular PGL.

##### 3.3.2. ‘Wait and Scan’ Approach

Four patients with unilateral tumors were allocated to this approach (Table 1) with annual MRI follow-up. Amongst patients with multiple HNPGLs, the youngest patient is being monitored without surgery due to the medical status of the patient and patient No. 26 declined all forms of treatment for the HNPGLs but requested facial reanimation only. Although he was kept under observation, the disease had already progressed considerably and he died from respiratory complications due to bilateral vagal palsy. Patient No. 28 declined any form of surgery and is therefore closely monitored. Furthermore, a similar method was adopted for non-operated tumors.

#### 3.3.3. Disease Control and Cranial Nerve Function Status on Last Follow-Up

All patients are followed up annually using MRI for anatomical evaluation and a functional imaging using PET/CT every 3 years. Status of cranial nerve function on last follow-up is given in Figure 2. Only the last patient in our series with *SDHD* mutation was diagnosed with a JPGL three years later on follow-up imaging. Even patients with *SDHB* mutation and the only one with the malignant tumor had no further progression of tumors or development. Cranial nerve functional status and tumor control has been achieved amongst our patients.

## 4. Discussion

Our cohort of patients showed higher female predominance (females: males = 1.7:1), but was less than expected prevalence (9). It has also been well-established that amongst all HNPGLs, carotid body tumors are the most common type (60% and more) [10], followed by jugulotympanic (<35–40%) and vagal (<5%) paragangliomas. We showed a total of 42 tumors with different distribution of CBPGLs (26.2%), JPGLs (31%), TPGLs (19%) and VPGLs (23.8%). This can be compared to a study done in Germany with large series of patients, where they showed highest frequency of JPGLs (39%) followed by CBPGLs (30.5%), TPGLs (14.3%) and VPGLs (11.7%) [27]. Other forms of head and neck paragangliomas were absent amongst our cohort. Additionally, only 1 out of our 6 patients with multiple HNPGLs had positive family history. A comparative study showed 20 out of 79 patients with multiple tumors where only 4 had positive family history [28]. Amongst 25 patients who underwent genetic examination, 3 had *SDHD* germline mutation and multiple tumors, commonly seen in HNPGLs [6], 1 out of 2 patients with positive family history had *SDHD* mutation. Risk of malignancy with *SDHB* mutation is higher [6]; however, in our series, amongst the 2 patients with *SDHB* mutation, one was histopathologically confirmed as benign and the other patient with multiple tumors has no clinical or radiological signs of metastasis.

The most common symptoms associated with our cohort of patients were hearing difficulties (53.3%), painless neck mass (33.3%), and pulsatile tinnitus (26.7%). Only 5 (16.7% of all) patients had different cranial nerve defects, interestingly, it did not include those with Shamblin II/III CBPGLs. This was relatively different in comparison to a study done on HNPGLs by the Mayo Clinic (34.1% neck mass; 5% decreased hearing) [12]. Five of our patients had incidental findings of HNPGLs, which is not an uncommon phenomenon on US imaging of the neck indicated for other neck pathology, especially the thyroid gland [6].

The investigative techniques used in our patients make up the current standard approach used in centers where patients with HNPGLs are managed [5,15,29]. Investigative techniques in paragangliomas help not only in diagnosing, but also in surveillance of tumors. In terms of imaging techniques, the availability of PET-CT has modernized and eased the comfort in which effective management can be based on [3]. 18F-FDOPA PET/CT, which was initially developed to investigate dopaminergic neurotransmission was found to be a highly sensitive (95% for HNPGLs) and specific (95–100%) imaging modality for the detection of PGLs, especially for HNPGLs [30]. 18F-FDOPA PET/CT may be performed for confirmation of diagnosis of pheochromocytoma/paraganglioma, staging at initial presentation, restaging, and follow-up of patients. It should be mentioned that for SDHx-mutated PPGLs, the practical use of 18F-FDG PET/CT turned out to be a revolution, with 100% and 92% detection rates for primary and metastatic tumor, respectively, leading to the 2014 recommendation by the US Endocrine Society Task Force for Pheochromocytoma, which stated that F-FDG PET/CT should be used for assessment of metastatic PPGLs [31,32,33]. In SDHx-related syndromes, it is recommended to use 18F-FDG PET/CT in addition to 18F-FDOPA PET/CT [31,32]. As part of biochemical investigations, apart from measuring catecholamines levels, which were normal in our cohort of patients even with hypertension, the tumor marker Chromogranin A was also analysed. In HNPGLs, Chromogranin A correctly predicts the result of paraganglioma surveillance more often in patients with *SDHB* compared with those with *SDHD* (77% vs. 22%, *p*  =  0.003) and has less added benefit to standard surveillance [34]. However, this test is also conducted to evaluate the risk of development of pheochromocytomas in such patients. It is a sensitive and specific diagnostic tool in detecting both familial and sporadic pheochromocytomas. Interestingly, the concentration of plasma chromogranin A also predicts the size of the pheochromocytoma [35]. Lastly, we should reiterate the importance of genetic examination; it is not only invaluable in predictability of tumor occurrence in index patients but also for surveillance in relevant at-risk relatives [3,4,5,6,7,8,26]. The most valuable application is predicting the risk of tumor development in multiple HNPGLs and/or familial forms of the disease where family history is negative [26]. The cost of genetic testing can vary from type of test and insurance coverage amongst different centers worldwide, the main disadvantage being the unavailability of specialized lab facilities in many countries.

Several approaches have been advocated in the management of HNPGLs [3]; the choice of treatment should be dependent on patient-related and tumor factors. Preventive, intermediate, definitive, and alternative management options for PGLs have been detailed in an earlier publication [26]. The two main aims of any treatment approach are long-term tumor control and minimal cranial nerve morbidity. Tumor control is defined as arrested tumor growth or shrinkage (partial or complete) of tumor.

HNPGLs are typically slow growing tumors (1–2 mm/year) [22,36], hence the ‘wait and scan’ approach can be used for asymptomatic cases with low risk of malignancy and ideally in the absence of germline mutation. These patients should be monitored very closely with regular MRI scans; otherwise, this always incurs a risk of irreversible complications from tumor infiltration.

If surgery is considered, a very important aspect of this technique is preoperative embolization; with improvements in preoperative preparation of patients, the expected risk of stroke has reduced from 30% to less than 3% and mortality rates are almost negligible during carotid surgery [37]. The risk of major vascular injury is very high for CBPGLs in Shamblin III [3]. We believe that preoperative embolization is not indicated in small tumors (e.g., carotid body Shamblin I and tympanic paragangliomas Fisch A). Preoperative embolization techniques including trans-arterial or direct puncture embolization of such tumors are performed either using resorbable materials like fibrin glue, gelfoam, polyvinyl alcohol, or permanent embolic agents such as ethanol [38,39]. The use of non-adhesive liquid agents such as Onyx (ethylene vinyl alcohol copolymers) has gained popularity due to a better rate of devascularization [40], as used for large tumors in our series. In more recent times, a study also reported the use of SQUID 12 in direct puncture of hypervascular tumors of the head and neck, which may offer almost complete devascularization, reduce intraoperative blood loss, and increase visibility to facilitate complete surgical resection of such tumors [41]. All our patients underwent preoperative embolization before surgery without post-interventional complications. Deterioration of cranial nerve status was observed in cases of nerve infiltration and encasement by the tumor.

Complete surgical resection of non-advanced cases is curative for HNPGL patients; however, size and localization should be respected. In our cohort of patients, 23 underwent surgery. We operated on 26 tumors (6 CBPGLs, 9 JPGLs, 5 TPGLs, 6 VPGLs), that is 61.9% of totally identified tumors.

We used the IFTA approach, which is well recognized as the best surgical approach for management of JGPGLs as it permits anterior and superior exposure of the jugular foramen. Anterior rerouting of CN VII is a crucial step in improving surgical exposure of jugular foramen with improved control of lower cranial nerves as well as petrous part of the internal carotid artery. Transient postoperative dysfunction of the facial nerve accompanies the manipulation of this nerve. In experienced hands, such dysfunction improves during the subsequent months, as demonstrated here. The highest incidence of cranial nerve deficits were seen amongst the patients who underwent surgery for JPGLs. A large study reviewed the results of 1084 JPGLs patients who underwent surgical resection. Long-term tumor control was obtained in 78.2% of patients, but a total of 1,183 preoperative cranial nerve palsies increased to 2,148 definitive postoperative cranial nerve palsies. Therefore, surgery actually led to 965 new cranial nerve deficits [42].

Selective neck dissection was performed both because the tumors are often associated with lymphadenopathy and proper exposure of tumor as well as visualization of critical neurovascular structures is improved in this manner; furthermore, the presence of lymph node metastases in these tumors cannot be excluded without histopathologic examination. In one of our patients with JGPGL, this step led to the identification of lymph node metastasis that was not suspected both on preoperative imaging and clinical examination. Therefore, this procedure carries a diagnostic purpose. Dysphagia associated with vagal nerve dysfunction seen postoperatively in our patients with VPGLs was well compensated functionally, additionally, vocal cord paralysis was absent in all these cases. Our results show that the long-term resolution of cranial nerve function was largely dependent on preoperative status. None of the patients had sympathetic trunk deficit.

Other studies showed the long-term results after complete surgical resection of CBPGLs to be excellent, with cure rates reported to be as high as 89% to 100%, well over 90% in VPGLs and 92.5% for tympanic tumors [42,43,44]. Over a period of 1 to 4 years depending on the time of surgery, we achieved local control in 22 out of 23 patients; 1 had residual tumor but with signs of shrinkage. Furthermore, tumor samples will be tested for the presence of somatic mutation in the near future.

In terms of patients with multiple HNPLs, if surgery is considered, the aim is always a stepwise approach, thus avoiding bilateral cranial nerve deficits and catastrophic disabilities [26]. It is recommended to remove the larger tumor first.

The second most common treatment strategy used for several decades is radiotherapy, on the basis that paragangliomas are radiosensitive. External-beam radiation technique carries a small risk of xerostomia, decreased hearing, alopecia, and stenosis of external auditory canal, among others [45]; hence, preference is given to radiosurgery. Image-guided radiosurgery or stereotactic surgery uses a more precise form of radiation with recent advances, having a low rate of side-effects with actuarial 10-year progression-free survival approaching 86.3–92% [45,46]. This approach has particularly gained popularity with JPGLs; lower cranial nerve dysfunction would be associated with large jugular tumors either preoperatively [47] or whilst achieving complete resection postoperatively [48]. In cases of JPGLs, high rates of cranial nerve injuries, incomplete resection, and aggressive behavior of these tumors advocate the use of preoperative radiotherapy [3]. Although comparative analysis showed clinical improvement in 30% of 142 patients who underwent Gamma Knife radiosurgery versus 374 patients who underwent microsurgical resection [49], the use of radiosurgery in pre-existing cranial dysfunction with large tumors (of more than 7 cm) or postoperatively still carries a risk of worsening of the symptoms [46]. The presence of pre-existing nerve palsy should be considered as a negative factor for both radiosurgery or surgical intervention. Similarly, stereotactic surgery also has reported disadvantage of size when treating CBPGLs; the considered ideal size is <3 cm. Furthermore, there is no sufficient evidence to justify the use of radiosurgery over surgical resection in cases of secretory HNPGLs [3]. The other consideration is the long-term risk of developing delayed radiation-induced malignancies when treating benign paragangliomas [45]. The incidence of radiation-induced fibrosarcoma is approximately 1 in 1000 to 2000 [50]. Other malignancies such as anaplastic astrocytoma [51], malignant peripheral nerve sheath tumor of the vagus nerve [52], and even brainstem glioblastoma have also been reported [53].The main problem is that such malignancies may rise a few decades after treatment; hence, it may not be ideal in young patients. Future developments in radiation would be treating multifocal tumors, thus allowing irradiation of 3 or 4 synchronous sites. Another disadvantage of using radiotherapy is unavailability of direct tumor sampling for genetic evaluation of somatic mutation, which in patients with both positive and negative germline statuses helps to plan for future treatment in case of recurrence or metastasis. When combined with the fact that there is emerging evidence that open surgical resection has a risk of cranial nerve injury thus reducing quality of life and an operative mortality of 1/100 [54,55,56], radiotherapy is increasingly considered as a frontline modality or postsurgical salvage. However, given the fact that many other factors also play a role in decision-making, we recommend to use this mode of treatment amongst patients with multiple HNPGLs to treat the contralateral side or as postsurgical salvage therapy.

Other advanced treatment modalities such as gene targeted therapy, therapeutic radiation, and radionuclide therapy [3] have some roles in metastatic disease and may be of significant value in the future due to versatile tumorigenic pathways of PGLs. In present times, detection of mutation type has a predictive value of disease control but does not offer any customized mutation-specific therapy.

## 5. Conclusions

Modern scientific medicine has significantly changed the outlook of management in HNPGLs. Over the decades, new diagnostic, therapeutic, and prognostic insights of these tumors have been discovered. Preventive management has emerged significantly. Therefore, tumor surveillance using genetic mutation analysis, whole body imaging, and biochemical testing plays a significant role not only in the management of this disease amongst index patients but also at-risk relatives of such cases. Younger age of presentation, multiple tumors, and positive family history should be strongly suspected of hereditary tumors; therefore, genetic testing is also highly recommended. Although only a fraction of HNPGLs are secretory, it is an important factor with respect to preoperative preparation of patient and options for definitive treatment. The ‘wait and scan’ approach has also been popularized due to the slow-growing nature of these tumors, but does carry the risk of irreversible damage from tumor infiltration. Size and localization of HNPGLs as well as preoperative cranial nerve status play very important roles in the decision for surgery. Multiple bilateral HNPGLs should never be operated in a single stage. Diagnosing malignancy in HNPGLs can be challenging in the absence of defined histomorphological criteria; therefore, lymph node dissection should be considered, even in the absence of clinical and radiological signs of metastasis. Preoperative embolization is strongly recommended for those undergoing surgery. We achieved tumor control in nearly 96% of our patients that underwent surgery with minimal cranial nerve dysfunction, hence reiterating that this technique with the right experience still plays a major role in the outcome of the disease. If necessary, radiosurgery can complement surgery to minimize comorbidities associated with surgery alone, but if being considered as frontline therapy, it must be used with caution in young patients or with hereditary tumors. In secretory tumors, this technique is inadvisable. Cranial nerve dysfunction in association with tumor encasement is a negative prognostic factor for both surgery and radiotherapy. Long-term follow-up should be done in all cases to facilitate the early detection of recurrent or new tumors and status of cranial nerve function.

## Figures and Tables

**Table 1:** Summary of patients with HNPGLs at our clinic between 2016 to 2020.

Pt.	Age (Years)	Gender	Medical History	F/H of PPGLs	Tumor(s)		Type of Treatment		
					Laterality	Classification			CBPGL
1	47	M	Asthma	-	R	Shamblin II	Surgery		JPGL
2	62	M	Hypertension	-	L	Shamblin II	Surgery		TPGL
3	64	M	Pangastritis	-	R	Shamblin II	Surgery		VPGL
4	76	F	-	+	R	Shamblin I	W + S		
5	37	F	Asthma, hypertension, renal angiomyolipoma	-	L	Fisch C3 Di2	Surgery		
6	37	F	-	-	L	Fisch C3	Surgery		
7	43	F	Hypertension, hydrocephalus	-	L	Fisch C3 Di3	Surgery		
8	54	F	Hypertension, diabetes mellitus	-	R	Fisch C3Di1	Surgery		
9	60	F	Hypertension	-	L	Fisch C3 De2	Surgery		
10	64	M	Diabetes mellitus	-	L	Fisch C3	Surgery		
11	69	F	Diabetes mellitus, Leiden mutation	-	R	Fisch C1	Surgery		
12	80	F	Hypertension	-	L	Fisch C3 Di3	W + S		
13	42	M	Hypertension, diabetes mellitus	-	L	Fisch B3	Surgery		
14	48	F	Hypertension	-	L	Fisch A1	Surgery		
15	48	F	-	-	R	Fisch B2	Surgery		
16	55	F	Hypothyroidism, duodenal ulcer	-	L	Fisch B2	Surgery		
17	57	M	-	-	L	Fisch C2 Di1	W + S		
18	67	F	-	-	R	Fisch C1	Surgery		
19	71	F	-	-	L	Fisch C1	W + S		
20	39	F	Asthma	-	L	Fisch A	Surgery		
21	44	F	Hypothyroidism	-	R	Fisch A	Surgery		
22	46	M	-	-	R	Fisch A	Surgery		
23	51	F	-	-	R	Fisch A	Surgery		
24	51	F	Hypertension, Migraine	-	L	Fisch A	Surgery		
25	34	M	Tetralogy Of Fallot	-	B	L: Shamblin III, R: Shamblin II	W + S		
26	36	M	Paranoid schizophrenia	-	B	Shamblin III	Treatment declined Deceased		
					R	Fisch C			
					R	Fisch C4 Di2			
27	43	F	Spontaneous abortion	+	R	Shamblin II	Surgery		
					L	Fisch A	W + S		
28	47	M	-	-	L	Fisch B	W + S		
					L	Fisch C1	W + S		
29	51	M	Hypertension	-	B	Shamblin II	Surgery		
					B	Fisch C1	Surgery		
					L	Fisch A1	W + S		
30	57	F	Hypertension, Hyperlipoproteinemia	-	R	Fisch A	Surgery		
					L	Fisch C1	W + S		

F/H of PPGLs: Family History of Pheochromocytomas and Paragangliomas; L: Left; R: Right; B: Bilateral; W + S: Wait and Scan.

**Table 2:** Mode of presentation amongst HNPGLs patients.

		Number of Patients [Total (N = 30)] *n* (%)
	Demographic profile	
Gender	Males	11 (36.7%)
	Females	19 (63.3%)
Age at presentation below 40 years		5 (16.7%)
Medical history of hypertension		11 (36.7%)
Patients with negative family history		28 (93.3%)
	Clinical features	
Asymptomatic		2 (6.7%)
Painless neck mass		10 (33.3%)
Pulsation in the neck		1 (3.3%)
Dysphonia/hoarseness of voice		3 (10%)
Dysphagia		3 (10%)
Dysarthria		2 (6.7%)
Facial nerve palsy		2 (6.7%)
Restrictive tongue movement Hearing difficulty Pulsatile tinnitus Pressure in the ear Otorrhagia		1 (3.3%)
		16 (53.3%)
		8 (26.7%)
		1 (3.3%)
		1 (3.3%)
Imaging studies		
Presence of HNPGLs as incidentalomas		5 (16.7%)
HNPGLs		
	Solitary	24 (80%)
	Multiple	6 (20%)
Presence of PGLs below the neck		3 (10%)
Presence of non-PGL tumors		6 (20%)

## Data Availability

Data will be made available on request.
